# The Role of APP in Structural Spine Plasticity

**DOI:** 10.3389/fnmol.2017.00136

**Published:** 2017-05-10

**Authors:** Elena Montagna, Mario M. Dorostkar, Jochen Herms

**Affiliations:** ^1^Department for Translational Brain Research, German Center for Neurodegenerative Diseases (DZNE), Ludwig-Maximilian-University MunichMunich, Germany; ^2^Center for Neuropathology and Prion Research, Ludwig-Maximilian-University MunichMunich, Germany; ^3^Munich Cluster of Systems Neurology (SyNergy), Ludwig-Maximilian-University MunichMunich, Germany

**Keywords:** APP, dendritic spines, synaptic plasticity, *in vivo*, d-serine

## Abstract

Amyloid precursor protein (APP) is a transmembrane protein highly expressed in neurons. The full-length protein has cell-adhesion and receptor-like properties, which play roles in synapse formation and stability. Furthermore, APP can be cleaved by several proteases into numerous fragments, many of which affect synaptic function and stability. This review article focuses on the mechanisms of APP in structural spine plasticity, which encompasses the morphological alterations at excitatory synapses. These occur as changes in the number and morphology of dendritic spines, which correspond to the postsynaptic compartment of excitatory synapses. Both overexpression and knockout (KO) of APP lead to impaired synaptic plasticity. Recent data also suggest a role of APP in the regulation of astrocytic D-serine homeostasis, which in turn regulates synaptic plasticity.

## Structural Plasticity

Structural synaptic plasticity refers to morphologically observable changes of synapses which accompany the classical electrophysiological events during synaptic plasticity. Most prominent among them are dynamic changes in the number and shape of dendritic spines, which correspond to the postsynaptic compartment of glutamatergic excitatory synapses. Dendritic spines are small (1–2 μm long) protrusions of the dendritic shaft, which receive excitatory synaptic input and compartmentalize calcium (Majewska et al., 2000; Yuste and Bonhoeffer, 2001; Yuste, 2011) and therefore dictate the biophysical characteristics of a postsynapse. They are fundamental players in establishing and maintaining the neuronal network as well as other complex functions such as learning and memory. Conventionally, dendritic spines are classified according to their morphology into three different groups: thin spines, which are fine and long but have a discernible head; stubby spines, with a large head and an indiscernible neck and mushroom spines with big head and thin neck (Yuste and Bonhoeffer, 2004; Alvarez and Sabatini, 2007; Herms and Dorostkar, 2016). Additionally, filopodia are very motile protrusions that can transform themselves into mushroom or thin spines (Alvarez and Sabatini, 2007). However, a STED and EM based study revealed a higher degree of heterogeneity of both spine size and morphology (Tønnesen et al., 2014). These morphologies reflect different functional properties: for example, thin spines are more dynamic and more plastic than mushroom and stubby spines, which are thought to be more stable (Yuste and Bonhoeffer, 2001; Knott et al., 2006). A fraction of spines are continuously retracted and newly formed, and this process, expressed as turnover rate (TOR), is accelerated during learning and memory formation (Fu and Zuo, 2011).

Dendritic spines were discovered by Ramon y Cajal, who used Golgi’s silver staining method to visualize dendrites and their processes (Yuste and Bonhoeffer, 2001). While essentially the same technical approach is still used today, modern research on spines is typically conducted on transgenic animals expressing a fluorophore in a sparse subset of neurons (Feng et al., 2000). This allows visualization of spines on confocal microscopes, and, more importantly, *in vivo* observation of the dynamic changes comprising structural plasticity.

## Amyloid Precursor Protein Is a Synaptic Protein

**Figure 1:**
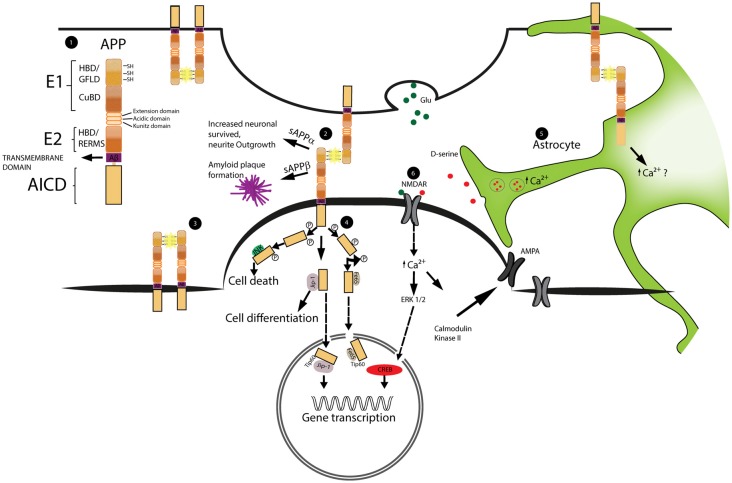
**Schematic representation of amyloid precursor protein (APP) structure and function at synapses.** The dimerization of APP as well as the signal cascade triggered by APP intracellular domain (AICD) are important for the regulation of spine stability. Astrocytes play a role in the regulation of spine dynamics via the calcium dependent release of the glio-transmitter D-serine. **1.** Schematic representation of APP domain structure. From the N-terminal region; the E1 domain formed by: heparin binding domain (HBD), growth factor like domain (GFLD) and cupper binding domain (CuBD). The E2 domain that includes the heparin binding domain and the pentapetide sequence (RERMS). Aβ region and transmembrane region precede the AICD intracellular domain. **2, 3.** Example of APP dimerization occurring at the synapses and between two molecules of APP on the same neuron. The dimerization is stabilized by the formation of disulfide bridges (SH-SH) highlighted in yellow. **4.** Schematic representation of AICD intracellular pathway. Phosphorylated AICD interacts with JNK triggering cell death, with JIP stimulating cell differentiation and with Fe65 or JIP to get transport into the nucleus and modulate gene transcription. **5.** Representation of astrocytic D-serine release. D-serine is stored inside vesicles. Upon increase of intracellular calcium these vesicles fuse with the cellular membrane releasing D-serine into the extracellular space. The precise role played by APP is still not clear **6.** D-serine together with Glutamate (Glu) activates NMDA receptors (NMDAR). NMDAR activation leads to the increase expression of AMPA receptors (AMPAR) on the membrane and triggers the activation of transcriptional factors into the nucleus.

Amyloid precursor protein (APP) is a member of a family of conserved type I membrane proteins which also includes APP like one protein (APLP1) and APP like two protein (APLP2; Wasco et al., 1992, 1993; Slunt et al., 1994). The major APP isoform expressed in neurons is 695 amino acids long, while longer isoforms are expressed in other tissues. Full-length APP consists of four main domains: the extracellular domains E1 (Dahms et al., 2010) and E2; a transmembrane sequence (Dulubova et al., 2004; Keil et al., 2004; Dahms et al., 2012); and the APP intracellular domain (AICD; Kroenke et al., 1997; Radzimanowski et al., 2008; Coburger et al., 2014; Figure 1). APP can be cleaved by a large number of proteases, which are grouped into α-, β- and γ-secretases, depending on the cleavage site. However, proteases which cleave APP outside these three sites also exist (Vella and Cappai, 2012; Willem et al., 2015; Zhang et al., 2015; Baranger et al., 2016). Depending on the combination of proteases which process APP, a vast number of different cleavage products may be generated, which have various biological properties (Nhan et al., 2015; Andrew et al., 2016). Among them are, for instance, amyloid β fragments which are generated by the action of β, and γ-secretases and which are known to be involved in the pathogenesis of Alzheimer’s disease. Other proteolytic products, such as the soluble fragment sAPPα and CTFs have been shown to be neuroprotective (Chasseigneaux and Allinquant, 2012; Hick et al., 2015; Andrew et al., 2016). Furthermore, *in vitro* evidence suggests that CTFs induce axonal outgrowth by interacting with G-protein αs subunits, which in turn activate adenylyl cyclase/PKA-dependent pathways (Copenhaver and Kögel, 2017), although these findings have not been corroborated *in vivo*.

In the brain, APP reaches its highest expression level during early postnatal development (from P1 to P36 in mice) and is preferentially localized at pre- and postsynapses (De Strooper and Annaert, 2000). During this period, synaptogenesis occurs and neuronal connections are formed (Hoe et al., 2009; Wang et al., 2009). Accordingly, many studies described putative roles of APP in the modulation of neurite outgrowth and synaptic connectivity (Moya et al., 1994; De Strooper and Annaert, 2000; Herms et al., 2004; Wang et al., 2009; Hoe et al., 2012; Müller and Zheng, 2012; Weyer et al., 2014; Hick et al., 2015). Synaptogenesis and neurite outgrowth may be mediated by full-length APP, which has been shown to exhibit cell adhesion- and receptor-like properties (Qiu et al., 1995; Ando et al., 1999; Turner et al., 2003; Soba et al., 2005; Müller and Zheng, 2012; Coburger et al., 2014; Deyts et al., 2016): there is convincing evidence that two distinct extracellular E1 domains from neighboring molecules of APP, APLP1 and APLP2 (Soba et al., 2005; Baumkötter et al., 2012; Deyts et al., 2016) can interact via their heparin binding domains (HBDs), and form a so-called heparin cross-linked dimer (Coburger et al., 2014). The interaction of the E2 domains with heparin cross-linked dimers further strengthens the dimerization process (Wang et al., 2009; Hoefgen et al., 2014). As APP is present both on pre- and postsynaptic terminals, a dimerization across the synapse may be relevant for synapse formation and stabilization (Wang et al., 2009; Baumkötter et al., 2014; Stahl et al., 2014). Moreover, the interaction of E1 and E2 domains with extracellular matrix components, like collagen, heparin, laminin, glypican, F-spondin and β1- integrin reinforces APP dimerization, and may further modulate the stability or plasticity of dendritic spines (Beher et al., 1996; Williamson et al., 1996; Rice et al., 2013; Wade et al., 2013).

Furthermore, growth factors and receptor-like proteins have been shown to interact with the APP-extracellular domains (Reinhard et al., 2005; Coburger et al., 2014; Deyts et al., 2016). Thus, activation of growth factor receptors could be an alternative mode of action of how APP affects spine plasticity. Additionally, the intra-cellular domain AICD itself may mediate receptor-like activity (Cao and Südhof, 2001, 2004; McLoughlin and Miller, 2008; Müller et al., 2008; Klevanski et al., 2015). Here, an intracellular response is triggered by the interaction of AICD-cleavage products with effector and adaptor proteins from the cytosolic compartment (Okamoto et al., 1990; Timossi et al., 2004; Deyts et al., 2012; Figure 1).

In addition to developmental processes, APP has also been shown to be involved in synaptic plasticity of mature synapses. For instance, some AICD-proteolytic products can be directly translocated into the nucleus and activate several transcription factors, like CP2/LSF/LBP1 or Tip60 (Müller et al., 2008; Schettini et al., 2010; Pardossi-Piquard and Checler, 2012), which are known to be involved in the regulation of dendritic spine plasticity.

## App Is Involved in Structural Spine Plasticity

Two main bodies of evidence support a role of APP in structural plasticity. On one hand, overexpression of APP, which is often used to model Alzheimer’s disease, may alter dendritic spines independently of typical Alzheimer’s disease pathology. These findings are described later in this section. On the other hand, knockout (KO) of APP alters spine dynamics: in the hippocampus, APP KO causes a range of synaptic alterations, depending on the model and paradigm studied. For instance, in cultured hippocampal neurons of APP KO animals, we found enhanced amplitudes of evoked AMPA- and NMDA-receptor-mediated EPSCs, which were reduced by pre-conditioned wildtype medium. Additionally, we found an increased density of synaptophysin-positive presynaptic puncta (Priller et al., 2006). The number of dendritic spines, in contrast was reduced (Tyan et al., 2012) in APP KO neurons, while it was increased in APP overexpressing neurons (Lee et al., 2010). In organotypic slice cultures APP-KO neurons showed a pronounced decrease in spine density and reductions in the number of mushroom spines, which was rescued by sAPPα expression (Weyer et al., 2014). These results suggest that soluble sAPPα modulates synaptic function in the neonatal hippocampus. A study in hippocampal slices of adult APP KO mice found decreased paired-pulse facilitation in the dentate gyrus, while granule cell excitatory transmission was unaltered (Jedlicka et al., 2012). These contrasting findings may be the result of region-specific differences in APP expression in the hippocampus (Del Turco et al., 2016).

**Figure 2:**
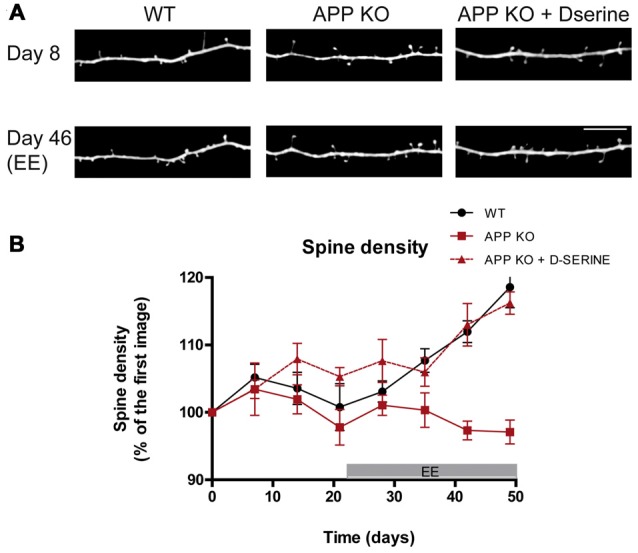
**Stimulation-induced synaptic plasticity is deficient in APP knockout (KO) mice and can be restored upon D-serine administration. (A)** *In vivo* images of apical dendrites from layer V pyramidal neurons in the somatosensory cortex of WT, APP KO and APP KO mice treated with D-serine, before and after exposure to enriched environment (EE), which broadly stimulates sensory and motor function. Scale bar, 10 μm. **(B)** Statistical summary of alterations in relative spine density over time. WT mice respond with increased spine density and turnover, while APP KO mice do not. Treatment with D-serine restores EE-induced synaptic plasticity in APP KO. (WT, *n* = 5; APP KO, *n* = 6; APP KO + D-serine, *n* = 4). Figured modified from Zou et al. (2016).

We recently studied dendritic spines of layer V pyramidal neurons of the somatosensory cortex in 4 month old APP-KO × GFP-M mice (Zou et al., 2016), which is accessible to chronic *in vivo* imaging. The density and the TOR of dendritic spines were monitored for a period of 9 weeks in comparison to GFP-M control mice (Figure 2). No differences were detected in the overall spine densities between the groups, whereas the fate of individual spines over time exhibited significant changes in their elimination and formation rates, resulting in reduced spine TOR (Zou et al., 2016). Since an alteration in spine plasticity is often correlated with alteration in spine morphology, we performed morphological analyses and found a decrease in the fraction of thin spines and an increase in the fraction of mushroom spines (Zou et al., 2016). These findings mirror the dynamic changes in TOR as thin spines are typically less stable than mushroom or stubby spines. In an earlier article (Bittner et al., 2009), in contrast, we had found an increased number of spines in APP KO, while turnover was not analyzed in detail. Two main factors may explain this apparent discrepancy: first, the data from the 2009 article were recorded almost a decade earlier, on an older generation multiphoton microscope. Modern microscopes have become considerably better at resolving thin spines. APP KO changes the morphology from thin to mushroom spines, which are more voluminous and thus easier to detect. Since thin spines used to be harder to detect, the results may have been interpreted as an apparent increase in spine densities. Second, the 2009 study used the YFP-H mouse line to label neurons, while the 2016 study used the GFP-M line. Although the populations of neurons which are labeled in both lines overlap, they are not identical. Thus, the subset of neurons analyzed in the earlier study may have had a different response to APP KO or it may have had a relatively higher fraction of thin spines, thus aggravating the first factor.

In order to understand whether the reduced TOR in APP-KO can be increased by physiological stimuli, we exposed APP KO mice to enriched environment (EE) which enhances the spine plasticity in several brain regions and increases TOR (Berman et al., 1996; Kozorovitskiy et al., 2005; Nithianantharajah and Hannan, 2006; Mora et al., 2007; Jung and Herms, 2014; Sale et al., 2014). However, APP KO mice exposed to EE for 5 weeks did not exhibit the physiological increase in spine density which was observed in WT controls (Zou et al., 2016). Thus, loss of APP leads to impaired adaptive spine plasticity (Figure 2).

In order to elucidate which domain of APP modulates dendritic spine plasticity, spine density and TOR were investigated in APP-ΔCT15 mice (Ring et al., 2007). These mice express a truncated form of APP, lacking 15 amino acids at the C-terminus, which correspond to the AICD. It was shown that several other phenotypes of APP-KO mice were rescued in APP-ΔCT15 mice, such as growth rates, brain weight, grip strength, locomotor alterations and spatial learning associated with long term potentiation (LTP) impairment in aged mice (Müller et al., 1994; Zheng et al., 1995; Dawson et al., 1999; Magara et al., 1999; Ring et al., 2007).

To further elucidate the role of APP in spine dynamics, our team conducted a study on 4–5 month old APP 23-GFP-M mice by 2-photon microscopy *in vivo*. APP 23-GFP-M mice overexpress human APP (isoform 751) with the Swedish (KM670/671NL) mutation under the murine Thy1 promoter (Sturchler-Pierrat et al., 1997). This leads to the formation of amyloid β deposits starting at 6 months of age and therefore this mouse line is considered to be a model of amyloidosis. However, our study revealed a significant decrease in dendritic spine density of layer V neurons of the somatosensory cortex (Zou et al., 2015) before the appearance of Aβ plaques, which was correlated with the amount of intracellular APP accumulating in neurons. Intracellular APP accumulation has been shown to mediate neuro- and synaptotoxicity in a number of publications (Neve et al., 1992; Fukuchi et al., 1994; Oster-Granite et al., 1996; Lu et al., 2003). Thus, it is crucial to distinguish between these different causes of synaptotoxicity when studying models of amyloidosis, as they do not all necessarily reflect human disease.

## App Regulates Spine Plasticity by Modulation of Astrocytic D-Serine

An additional mechanism for APP-mediated spine-arrangement is suggested by its modulation of astrocytic D-serine homeostasis, which is a modulator of synaptic NMDA receptors (Engert and Bonhoeffer, 1999; Hering and Sheng, 2001; Lai and Ip, 2013). The calcium-dependent astrocytic release of D-serine modulates NMDA-dependent LTP (Henneberger et al., 2010). It has been shown that full-length APP and its fragments modulate D-serine secretion (Wu and Barger, 2004; Wu et al., 2007) as well as astrocytic calcium homeostasis (Hamid et al., 2007; Linde et al., 2011). More recently, biosensor measurements in the cortex of 4–6 month old APP KO mice revealed decreased extracellular D-serine levels, while total D-serine was increased (Zou et al., 2016). These results suggest an alteration of D-serine homeostasis in APP deficient mice may underlie the altered regulation of spine dynamics. Treatment with exogenous D-serine for 5 weeks, supplemented in drinking water of standard housed and EE mice, restored extracellular D-serine levels and normalized the concentrations of total D-serine and L-serine in APP-KO brain (Zou et al., 2016). Furthermore, the administration of D-serine rescued the impaired dendritic structural plasticity in APP-KO mice: D-serine treated APP-KO mice had restored spine dynamics under standard housing conditions. Moreover, upon environmental enrichment, the fraction of thin spines was enhanced, while fraction of mushrooms spines was decreased (Figure 2). Although these data do not contest the synaptic role played by APP, they suggest a new interaction between APP and the D-serine homeostasis which is involved in spine dynamics and plasticity.

## Conclusions

Several mechanisms by which APP may modulate spine plasticity have been identified (summarized in Figure 1): structural properties of the full-length protein may help stabilizing synapses, while binding of ligands to the extracellular part may trigger intracellular cascades, similar to a classical receptor molecules. Additionally, recent findings demonstrate that APP modulates astrocytic D-serine homeostasis, which interacts with NDMA receptors to modify synaptic function. Lastly, neurotoxic and neuroprotective APP fragments may trigger or alleviate pathophysiological mechanisms involved in neurodegenerative diseases. Thus, APP seems to regulate synaptic plasticity at several levels. Yet, the relative importance of each of these mechanisms in physiology and disease remains to be elucidated.

## Author Contributions

EM and MMD wrote the review article, prepared figures. JH wrote the review article.

## Conflict of Interest Statement

The authors declare that the research was conducted in the absence of any commercial or financial relationships that could be construed as a potential conflict of interest.
